# Manipulation of the Cellular Membrane-Cytoskeleton Network for RNA Virus Replication and Movement in Plants

**DOI:** 10.3390/v15030744

**Published:** 2023-03-14

**Authors:** Rongrong He, Yinzi Li, Mark A. Bernards, Aiming Wang

**Affiliations:** 1 London Research and Development Centre, Agriculture and Agri-Food Canada, 1391 Sandford St., London, ON N5V 4T3, Canada; 2 Department of Biology, University of Western Ontario, 1151 Richmond St. N., London, ON N6A 5B7, Canada

**Keywords:** plant virus, positive-sense RNA virus, virus replication, endomembrane, cytoskeleton, virus intercellular movement, virus-plant interaction

## Abstract

Viruses infect all cellular life forms and cause various diseases and significant economic losses worldwide. The majority of viruses are positive-sense RNA viruses. A common feature of infection by diverse RNA viruses is to induce the formation of altered membrane structures in infected host cells. Indeed, upon entry into host cells, plant-infecting RNA viruses target preferred organelles of the cellular endomembrane system and remodel organellar membranes to form organelle-like structures for virus genome replication, termed as the viral replication organelle (VRO) or the viral replication complex (VRC). Different viruses may recruit different host factors for membrane modifications. These membrane-enclosed virus-induced replication factories provide an optimum, protective microenvironment to concentrate viral and host components for robust viral replication. Although different viruses prefer specific organelles to build VROs, at least some of them have the ability to exploit alternative organellar membranes for replication. Besides being responsible for viral replication, VROs of some viruses can be mobile to reach plasmodesmata (PD) via the endomembrane system, as well as the cytoskeleton machinery. Viral movement protein (MP) and/or MP-associated viral movement complexes also exploit the endomembrane-cytoskeleton network for trafficking to PD where progeny viruses pass through the cell-wall barrier to enter neighboring cells.

## 1. Introduction

Viruses are intracellular biotrophic parasites that infect all living organisms and exclusively replicate themselves in the infected cells. The majority of viruses that infect animals and plants are positive-sense single-stranded (+ss) RNA viruses [1]. Plant-infecting +ssRNA viruses usually have a small genome with very limited coding capacity and rely on the host cellular machinery to fulfill their infection cycle [2,3,4,5]. Upon entry into host cells, the first step for +ssRNA viruses to establish their infection is to translate the viral genome. Subsequently, viral membrane protein(s), along with their interacting proteins, target and remodel cellular membranes to form viral replication organelles (VROs), unique intracellular membranous structures for viral genome replication [2,6,7,8,9]. These virus-induced membranous VROs, also known as viral replication complexes (VRCs), virosomes, virus inclusions, virus factories, or viroplasms, are believed to confine the viral replication process to a specific safeguarded microenvironment that protects the replicating and progeny viruses from being targeted by host antiviral responses such as RNA silencing. VROs contain the viral RNA template, replication-required viral proteins, and various host factors. The membranous origin, size, and shape of VROs may vary from virus to virus. Some VROs can be motile [10,11,12]. Some VROs may associate with plasmodesmata (PD) to facilitate viral intercellular transport. It is well-established that the subcellular formation and movement of VROs require the host cellular endomembrane system and the associated cytoskeleton [13].

## 2. Plant Endomembrane Organization and Cytoskeleton-Dependent Intracellular Transport

The plant endomembrane system consists of the endoplasmic reticulum (ER), Golgi apparatus, vacuole, nuclear envelope, plasma membrane (PM), trans-Golgi network/early endosome (TGN/EE), and prevacuolar compartment/multivesicular body/late endosome (PVC/MVB/LE) and transport vesicles [14]. As the largest organelle in the cell, the ER functions as the transportation system of proteins and other molecules. The ER is also the site for calcium storage and lipid biosynthesis, as well as protein synthesis, folding, modification, and assembly [15]. The ER is composed of flattened sheets, studded with ribosomes, and branched tubules. These components interconnect and form a highly dynamic, continuous membrane network that distributes throughout the whole cell, extending from the outer membrane of the nuclear envelope (termed as the nuclear ER, nER) to the PM. The ER can connect with other membrane-bound organelles directly through membrane contact sites (MCSs), including mitochondria, endosomes, peroxisomes, and the PM [16]. In plants, the ER-derived desmotubules extend through the central canal of PD to connect adjacent cells [17]. The Golgi apparatus, also known as the Golgi complex or Golgi body, functions as a factory responsible for modifying, sorting, and packaging proteins and lipids into vesicles for delivery to their targeted destinations. A plant cell may contain up to several hundred Golgi stacks [18]. They are highly mobile and move on the microfilaments and ER tubules [19]. Each Golgi apparatus consists of several stacked cisternae, including the cis-, medial- and trans-cisternae. Secretory proteins synthesized in the ER exit the ER-Golgi intermediate compartment in coat protein complex II (COPII)-coated vesicles, enter the Golgi apparatus at its cis-cisterna, pass through medial- and trans-cisternae, and then reach the TGN en route to multiple final destinations [18,20]. The TGN is a central sorting hub for secretory and endocytic trafficking [20,21]. It functions as the early endosome to receive endocytic materials from the PM or extracellular space, and as a sorting station that releases transport carriers such as secretory vesicles and clathrin-coated vesicles (CCV) to the PM or PVC [21,22]. PVC/MVB/LE serve as intermediate compartments between the TGN and the vacuole, and enable proteins to recycle before fusion with the vacuole [23]. The vacuole is responsible for degradation and waste storage, equivalent to animal lysosomes, and acts as a large storage compartment for proteins [24].

The plant endomembrane system controls the secretion of biomolecules and mediates the uptake of substances from the exterior of the cell and the delivery to specific intracellular locations [25]. The secretory and endocytic pathways are two major transport routes of the plant endomembrane system [14]. The newly synthesized proteins traffic from the ER via Golgi to the PM or the extracellular space, which is defined as the secretory pathway [26,27]. Some plant secretory proteins can follow alternative unconventional protein secretion routes, bypassing the Golgi apparatus [27,28]. Endocytosis is a major route for the entry of proteins, lipids, and extracellular materials into the cell via a series of endosomal compartments, and plays an essential role in cell-to-cell communication and cellular responses [29]. The membrane trafficking process in both secretory and endocytosis pathways requires budding from the donor membrane, tethering, and fusion to the target membrane [30].

In plant cells, the endomembrane compartments interact extensively with the cytoskeleton system that is composed of actin filaments (AFs), microtubules (MTs), and associated proteins [31,32]. The cytoskeleton provides cells with mechanical support, and restructures to enable essential biological processes such as cell division, growth, mobility, and intracellular transport. Cytoskeletal AFs and MTs undergo dynamic cycles of polymerization and depolymerization [33,34]. The severing activity of the actin depolymerizing factor (ADF)/cofilin family contributes to filament disassembly [35]. The architecture and dynamics of the filament array are regulated by actin-binding proteins [35]. Both AFs and MTs function as suitable tracks for their respective molecular motors, dynein or kinesin proteins for MTs, and myosin proteins for AFs. These motors carry cargo between intracellular compartments along the cytoskeleton [33]. Different from mammalian cells, plant cells use AF cables rather MTs as the primary track for long-distance intracellular transport [35]. In the plant system, AFs are involved in the delivery of secretory vesicles, the recovery of integral membrane proteins through endocytosis, and driving membrane bending, and, thus, are essential for organelle movement and positioning of membrane-bound compartments within cells [35,36,37]. For example, the structural and dynamic behavior of the ER network is regulated by the tightly associated AF and myosin class XI motor proteins [31,38,39]. The cortical array of MTs intersects with the ER-AF network (cMERs) and contributes to the stabilization and structure of the ER-AF network [40,41].

## 3. Plant Viruses Remodel Endomembrane Structures for the Formation of VROs

Upon infection, plant viruses preferentially target and remodel specific membranous organelles, such as the ER, peroxisomes, mitochondria, chloroplasts, and/or tonoplasts for the formation of VROs. In addition to the origin of membranes, the shape and size of VROs may also vary from virus to virus. Some viruses may not have strict requirements for a particular type of membrane source, as they can change to alternative organelles for replication [42,43,44]. The newly advanced electron tomography and light microscopy techniques provide more details on the architecture of VROs [45,46,47]. Along with substantial advances in the ultrastructure of the membrane-bound viral replication compartments, recent results have shed light on the role that viral and host factors play in rearranging these membranes.

### 3.1. Architecture and Membranous Origins of VROs

As mentioned above, different viruses target different organelles to form VROs. Based on their morphology, VROs may be divided into two groups: the invaginated/spherule type, and the protrusion type [48,49,50]. The former is characterized by the generation of invaginated spherules with neck-like channels that connect the interior of the spherule to the cytoplasm. The latter is developed through bending the donor membrane into the cytoplasm. The protrusion-type VROs can be single-membrane vesicles (SMVs), double-membrane vesicles (DMVs), or more complex forms such as MVBs. 

The ER is the most frequently targeted organelle to form VROs for viral genome replication. Viruses, such as *Brome mosaic virus* (BMV, the genus *Bromovirus*), *Tobacco mosaic virus* (TMV, *Tobamovirus*), *Red clover necrotic mosaic virus* (RCNMV, *Dianthovirus*), and *Cucumber leaf spot virus* (CLSV, *Aureusvirus*) induce membrane invaginations towards the ER lumen to form spherules or vesicles in the perinuclear region, or randomly dispersed in the cytoplasm [51,52,53,54,55]. The interior of these spherules is connected with the cytoplasm via a narrow neck-like structure. In the case of *Beet black scorch virus* (BBSV, *Betanecrovirus*), three-dimensional electron tomographic analysis reveals the formation of multiple ER-originated vesicle packets (VPs), which enclose anywhere between a few and hundreds of 50–70-nm independent invaginated spherules [45]. In plant cells infected by potyviruses such as *Turnip mosaic virus* (TuMV), convoluted membrane (CM) structures appear in close proximity to the rough ER, followed by the production of both SMV- and DMV-like structures [47,56]. These vesicle-like structures are in fact tubules [47]. *Peanut clump virus* (PCV, *Pecluvirus*) remodels the ER and induces the formation of MVBs in tobacco protoplasts [57]. Remarkably, *Wheat yellow mosaic virus* (WYMV, *Bymovirus*) remodels the ER to form membrane inclusion bodies (MIBs) [58], lamella-membranous bodies (MBs), and tubule-MBs [59]. 

Peroxisomes are preferred by some tombusviruses such as *Tomato bushy stunt virus* (TBSV) and *Cucumber necrosis virus* (CNV) to form VROs [46,60,61]. TBSV infection induces the progressive inward vesiculation of the pre-existing peroxisomal boundary membrane to form MVBs, resulting in the organelle’s interior housing up to several hundred spherical to ovoid vesicles 80–150 nm in diameter [62]. These spherules, located in the lumen of the MVB, have necks that connect them to the MVB boundary membrane. However, some other TBSV-related viruses, such as *Melon necrotic spot virus* (MNSV, *Carmovirus*) and *Carnation Italian ringspot virus* (CIRV, *Tombusvirus*), target and remodel mitochondria for VRO formation [63,64,65]. In MNSV-infected cells, the mitochondrial ultrastructural changes include dilated cristae and a vesiculated outer membrane. The altered mitochondria contain large internal interconnected dilations that appear to be linked to the cytoplasm through pores or complex structures. There are numerous SMVs (45–50 nm in diameter) along the external mitochondrial membrane and in the lumen of the large dilations. These vesicles are bottle-shaped and connected with the cytoplasm or the dilation lumen through neck-like structures. These abnormal mitochondria provide sites for MNSV replication, as MNSV RNA, dsRNA, and capsid proteins reside in the large dilations. Moreover, these abnormal organelles are frequently located close to lipid droplets, the ER, and plasmodesmata (PD) [64]. Interestingly, some tombusviruses have the ability to target alternative organelles for replication. In the yeast mutant in which the ER membrane is expanded or peroxisomes are absent, the TBSV replication site switches from peroxisomes to the ER [42,46]. TBSV can also utilize the purified yeast ER and mitochondrial membranes for replication in vitro [66]. Consistently, chimeric hybrid tombusviruses between CIRV and *Cymbidium ringspot virus* (CymRSV) that carry mitochondrial or peroxisomal targeting signals are able to form VROs in the newly targeted organelles [67]. 

Chloroplasts are an organelle unique to the plant cell and some protists. Many viruses, such as *Turnip yellow mosaic virus* (TYMV, *Tymovirus*), *Barley stripe mosaic virus* (BSMV, *Hordeivirus*), and *Tobacco rattle virus* (TRV, *Tobravirus*), build their VROs on chloroplasts [68]. In TYMV-infected cells, chloroplasts become swollen, rounded, and clumped together. Many chloroplasts develop numerous 50–60-nm vesicles along their peripheries via invagination of both inner and outer envelope membranes, and these spherules have an open channel connecting the interior of the vesicle to the cytoplasm [69]. During BSMV infection, outer membrane-invaginated spherules and large cytoplasmic invaginations (CIs) are induced in the chloroplast. The spherules are connected to the cytoplasm via a neck-like structure. The CIs are composed of packets (average diameter 112 nm) derived from the inner chloroplast envelope. Within the packet, there are various numbers of outer membrane-derived spherules (~50 nm in diameter). These spherules also have a neck-like structure open to the interior of the CI. These spherules contain viral RNA and replication proteins. The double membrane-bound CIs contain large amounts of BSMV virions. These CIs have various opening sizes connecting the CIs to the cytoplasm [70]. Thus, BSMV genome replication and virion assembly take place in these modified membranous structures derived from chloroplasts. In addition, potyviruses may initiate the VRO on the ER and subsequently target chloroplasts, and induce chloroplast amalgamation and envelope invaginations for robust viral replication [71,72].

The tonoplast, the semipermeable membrane surrounding the vacuole, is also targeted for viral replication by several plant viruses, such as *Cucumber mosaic virus* (CMV, *Cucumovirus*) or *Tobacco necrosis virus A* Chinese isolate (TNV-AC, *Alphanecrovirus*) [73,74]. Infection by CMV induces the invagination of the tonoplast and MVBs for the formation of 50–70-nm spherules. These spherules have pore-like openings toward the cytosol and are thought to function as VROs for viral replication [74]. 

**Figure 1:**
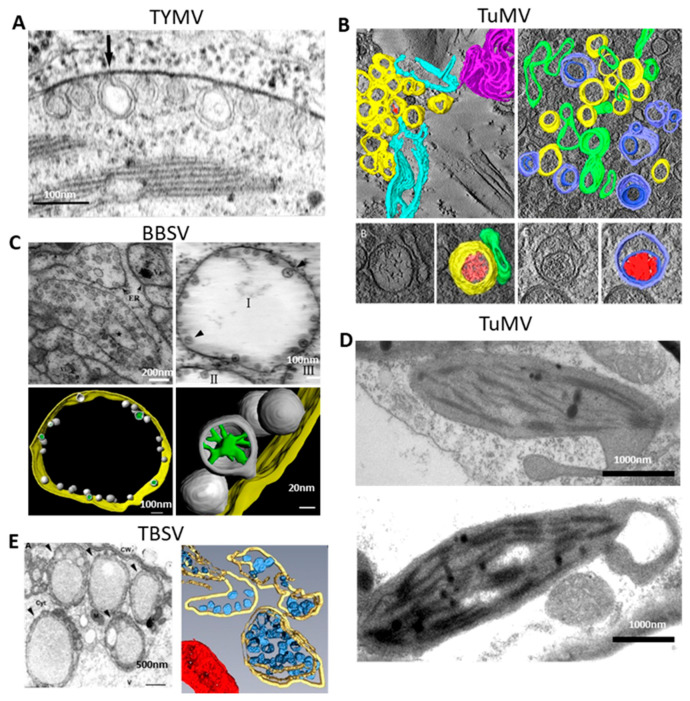
Virus-induced membranous structures derived from organellar membranes. (**A**) Electron micrograph of an ultrathin section of part of a chloroplast from TYMV-infected Chinese cabbage leaf cell. The arrow points to a vesicle in which an open channel is connecting the interior to the cytoplasm. Adapted from [69]. (**B**) Three-dimensional reconstruction of TuMV-induced membrane rearrangement. Overview of tomogram slice from TuMV-infected vascular parenchymal cell at the mid stage (upper left) and late stage (upper right) of infection. Three-dimensional model of a single-membrane tubule (SMT) with fibrillary material inside and with an adjacent intermediate tubular structure (lower left). Three-dimensional model of a double-membrane tubule (DMT) with a core of electron-dense materials inside (lower right). Yellow, SMTs; light red, electron-dense materials; green, intermediate tubular structures; light blue, outer membranes of DMTs; dark blue, inner membranes of DMTs; dark red, the electron-dense materials inside DMTs; sky blue, rough ER; magenta, cytoplasmic inclusion body; red arrow, connection between the rough ER membrane and an SMT. Adapted with permission from [47]. (**C**) Transmission electron microcopy (TEM) analysis and 3D model of BBSV-induced ER membrane rearrangements. (upper left) BBSV-induced vesiculation of the ER. Vesicle packets were observed in the aggregates of branched ER cisternae (*). Black arrowheads, ER membrane; Vi, virus crystal. (upper right) Tomographic slice of BBSV-induced vesicle packets and ER-derived spherules. I II and III represent different units of VPs. (Lower left) Three-dimensional model of BBSV-induced vesicle packets and ER-derived spherules. (Lower right) Enlargement of the connections between the spherules and the outer ER membrane. Gold, outer ER membrane; gray, BBSV-induced spherules; green, fibrillary materials inside the spherules. Adapted with permission from [45]. (**D**) TEM analysis of abnormally distorted chloroplasts in TuMV-infected leaves. (Upper panel) Chloroplast with membrane-bound extrusion. (Lower panel) Amoeboid shaping of chloroplasts, showing the extrusion of chloroplast encircling a large vesicle. Adapted with permission from [71]. (**E**) Electron analysis and 3D model of TBSV replication platform. (Left) Transmission electron micrograph of a TBSV-infected *N. benthamiana* leaf cell. Arrowheads, TBSV-induced individual peroxisomal multivesicular bodies (MVBs). Adapted with permission from [62]. (Right) Three-dimensional model of TBSV replication platform in yeast showing peripheral MVB with characteristic spherules. Yellow, peroxisome membrane; blue, spherules; red, mitochondrion. Adapted with permission from [46].

Taken together, different plant RNA viruses have their preferred organelles for VRO formation, but at least some of them seem to have the ability to utilize alternative cytoplasmic membranes for viral genome replication. A few examples of modified membranous structures are presented in Figure 1. 

### 3.2. Viral Proteins That Target and Remodel Host Organelle Membranes for VRO Biogenesis

VRO biogenesis is initiated when newly translated viral proteins target the preferred organelle(s) and remodel the organellar membrane. These viral proteins are usually membrane-associated proteins [6,75]. The transient ectopic expression of these viral proteins in noninfected cells is sufficient to induce VRO-like membranous structures similar to those observed in virus-infected cells. However, in some cases, the remodeled membrane structures by these viral proteins in the absence of the virus differ from those observed in virally infected cells, indicating that an intricate network of factors and conditions is required for proper membrane modifications [76]. These viral proteins may alter membrane shape directly, by associating with membranes to induce curvature, or indirectly, by cellular factors they recruit.

Many of these viral membrane-associated proteins have one or several transmembrane domains (TMDs) which are typically 12–35 residues (mostly hydrophobic amino acids). The TMD(s) are required for membrane insertion [62,77,78]. Some of these TMD proteins also contain amphipathic helices (AHs). In contrast, some other viral membrane-associated proteins do not contain a TMD and have one or multiple AHs, which are responsible for membrane association. Essentially, the AH protein has one polar and one hydrophobic side and lies flat on the membrane with the hydrophobic side embedded into the membrane, driving membrane destabilization and bending [79,80,81]. A large number of these AH proteins are able to oligomerize, which allows the helices to traverse the membrane by creating an aqueous pore [79,81].

Since the late 1990s, membrane proteins encoded by many plant RNA viruses, such as 6K2 from potyviruses, the nucleotide triphosphate-binding protein (NTB) of *Tomato ringspot virus* (ToRSV, *Nepovirus*), BMV 1a, and the p33 and p36 proteins from tombusviruses, have been studied. The potyviral 6K2 protein is an integral membrane protein and expression of 6K2 alone induces ER proliferations and the formation of numerous cytoplasmic vesicles [78,82]. The 6K2 protein contains a central hydrophobic α-helix domain of 19 amino acids flanked by charged residues. This domain is required for vesicle formation [78].

In the case of ToRSV, the NTB-VPg polyprotein, which is the precursor of NTB and VPg (viral protein genome linked) is associated with ER-derived membranes active in virus replication [81,83]. The NTB protein contains a TMD at the C-terminus, an N-terminal AH, and a central region with NTB activity. The AH is initially parallel to the membranes and the C- terminal TMD traverses the membrane [81]. Oligomerization of these elements results in the formation of aqueous pores and the translocation of the N-terminal AH with its hydrophilic faces toward the pore, and the hydrophobic faces interact within the membrane environment [81]. 

Different from 6K2 and NTB, the BMV replication protein 1a does not contain any TMD and instead has two AHs (AH A and AH B, 18 residues each) that are adjacent to each other. BMV 1a is a multifunctional protein and consists of a capping domain at the N-terminus and a helicase-like domain with NTPase activity at the C-terminus. AH A and AH B are located in the region E (114 amino acids in length) between the capping and helicase-like domains. Previous studies have suggested that the region E is necessary for nER membrane association, and AH A plays a role in nER targeting and regulates the size and frequency of VROs [84,85]. A more recent study has shown that it is AH B that is primarily responsible for nER targeting [86]. Similar to BMV 1a, RCNMV p27 has an AH in the N-terminal region which is essential for association with the ER [87].

For tombusviruses, such as TBSV and CIRV, viral proteins p33 and p36 that contain an N-terminal hydrophilic region and two TMDs are required for the membrane association of VROs. TBSV and CIRV target peroxisomes and mitochondria, respectively, to induce the formation of multivesicular bodies (MVBs). The N-terminal hydrophilic region and the TMDs specify which organelle is involved in VRO biogenesis [62,67]. In the case of MNSV, a TBSV-related virus, it is the p29 protein that has three TMDs and targets mitochondria to induce the disorganization and proliferation of mitochondrial membranes in a specific manner [64].

### 3.3. Host Factors Recruited for Membrane Modifications

While viral membrane-association proteins are critical for membrane modification, VRO biogenesis requires various host factors such as lipids, cytoskeleton, membrane-shaping proteins, and components of the membrane trafficking pathway.

#### 3.3.1. Membrane-Shaping Proteins

In high eukaryotes, at least four curvature-generating and -stabilizing protein families, including the Bib-Amphiphsin-Rvs (Bar) domain proteins (Bars), the reticulons (RTNs), the membrane-fusing GTPase atlastin (ATL), and the lunapark protein (Lnp), are known to affect the shape of membranes. Among them, BARs, RTNs and ATLs have been shown to be implicated in VRO biosynthesis [5,7]. BARs are involved in bio-membrane remodeling to induce membrane curvature and function in many membrane trafficking pathways. BARs have been shown to participate in the formation of spherule (vesicle-like) structures of tombusvirus VROs [5]. RTNs contain hydrophobic domains occupying the outer leaflet of the phospholipid bilayer. The curvature of the ER membrane results from the hydrophobic wedging, and possibly the scaffolding and protein-protein crowding formed by RTN oligomers [88]. The RTN homology proteins (RHPs) have been shown to be involved in the assembly of the ER-derived spherules during BMV infection. BMV 1a interacts with RHPs and relocalizes RHPs from peripheral ER tubules to the interior of the spherules. In addition, deleting RHPs prevents spherule formation and inhibits BMV RNA replication [89]. A recent report has identified a soybean RHP that interacts with the P3 protein of *Soybean mosaic virus* (SMV, *Potyvirus*) and contributes to viral replication. However, it remains unclear how RHPs are involved in the formation of potyviral VROs [90]. ATLs are large dynamin-related GTPases that mediate the tethering and fusion of tubules to form three-way junctions. In the mammalian cells infected by *Dengue virus* (DENV, *Flavivirus*), depletion of ATL2 alters the formation of ER-derived VROs by distorting vesicle size and shape and condensing the vesicles to a small perinuclear region [91]. In plants, the ATL-like GTPases ROOT HAIR DEFECTIVE3 (RHD3) plays an important role in the generation of the interconnected tubular ER network [92,93,94]. The ER-fusogen RHD3 has been demonstrated to be required for the maturation of TuMV viral replication vesicles [95].

The endosomal sorting complexes required for transport (ESCRT) machinery, made up of cytosolic protein complexes, known as ESCRT-0, ESCRT-I, ESCRT-II, and ESCRT-III, play a vital role in a number of cellular processes, such as MVB biogenesis and autophagy, through a unique mode of membrane remodeling that results in membrane bending/budding away from the cytoplasm [96,97,98]. During VRO biogenesis, ESCRT-mediated membrane deformation leads to the formation of spherule structures. For example, TBSV p33 interacts with vacuolar protein sorting-associated protein 23 (Vps 23; an ESCRT-I protein) and Bro 1 (an ESCRT accessory factor) to recruit Vps23 to TBSV replication sites, followed by the recruitment of ESCRT-III proteins (Snf7 and Vps24) and Vps4 AAA+ ATPase, which assist in the optimal assembly of VROs by deforming the membrane or stabilizing the neck structure of the spherules. Expression of dominant-negative mutants or deletion of ESCRT factors inhibits tombusviral replication [99,100,101,102]. Similarly, BMV also takes advantage of ESCRT factors for proper spherule formation. BMV 1a interacts with Snf7p and relocalizes with other ESCRT proteins to BMV replication sites. Deleting Snf7 inhibits viral replication and abolishes spherule formation. Unlike TBSV, BMV spherule formation does not require the ESCRT-I factor [103].

#### 3.3.2. Early Secretory Pathway Proteins 

The early secretory pathway is composed of the ER, Golgi bodies, and vesicles that travel between them [104]. The transport between ER and Golgi is bidirectional. Proteins that are properly folded in the ER are transported to the Golgi via COPII vesicles, while the Golgi-ER retrograde trafficking is mediated by COPI vesicles [104]. COPI or COPII vesicles or proteins promoting their formation have been implicated in the replication of many plant RNA viruses. ADP ribosylation factor 1 (Arf1), a small GTPase, regulates the formation of COPI vesicles [105,106]. Dimerization of Arf1 is critical for positive membrane curvature during the formation of coated vesicles [105,107]. Another small GTPase, secretion-associated Ras-related GTPase 1 (Sar1), is part of COPII and, upon GTP binding, recruits the other COPII proteins to the ER [108]. The SNARE (soluble N-ethyl-maleimide-sensitive-factor attachment protein receptor) complex mediates the membrane fusion of COPII- and COPI-vesicles with their target membranes [109].

The early secretory pathway has been shown to make essential contributions to VRO biosynthesis for diverse plant +ssRNA viruses. RCNMV p27 interacts with Arf1 in the virus-induced structures originating from the ER membrane. Inhibition of Arf1 using the inhibitor brefeldin A (BFA) disrupts p27-mediated ER remodeling and VRO assembly, and expression of a dominant-negative mutant of Arf1 or Sar1 compromises RCNMV RNA replication [110]. These findings suggest that remodeling of ER membranes by RCNMV requires the host membrane transport machinery. Biogenesis of potyviral replication vesicles occurs at ER exit sites (ERESs) in a COPI- and COPII-dependent manner [71,82]. Disruption of the COPI or COPII machinery by coexpression of dominant mutants of Arf1 and Sar1 inhibits the formation of virus-induced vesicles and suppresses viral infection [82]. Similar to 6K2, WYMV P2 protein is an integral membrane protein that depends on the active secretory pathway to form membranous compartments for replication [58]. P2 interacts with the COPII protein Sar1 to rearrange ER membranes into aggregate structures, and treatment with BFA or coexpression of a dominant-negative mutant of Sar1 inhibits the formation of aggregate structures [58]. In the case of BMV, a cargo receptor of COPII vesicles named 14-kDa ER-vesicle protein (Erv14) interacts with 1a [111]. This interaction targets BMV 1a to, or maintains BMV 1a at the perinuclear ER. Deletion of Erv14 disrupts the proper distribution of BMV 1a, leading to the BMV-induced spherules being less abundant in number but larger in size, and as a result, BMV RNA replication is significantly inhibited [111]. Expressing dysfunctional COPII coat proteins such as Sec13, Sec 24, or Sec 31 also disrupts the perinuclear ER localization of BMV 1a [111]. 

In the case of TBSV, a screening of Legionella effectors for antiviral effects led to the discovery that TBSV hijacks Rab1 (a small GTPase) and COPII vesicles to create enlarged membrane surfaces and optimal lipid composition within VROs [112]. Rab1 is a molecular switch that regulates vesicle traffic through different effectors. Rab1 binds to p33 and facilitates the recruitment of COPII vesicles into VROs. Interference with the recruitment of Rab1 or subversion of COPII vesicles prevents the formation of regular-sized VROs [112]. The efficient recruitment of Rab1 into TBSV VROs depends on Syntaxin 18-like Ufe1 and Use1, which are components of the ER-resident SNARE complex in the ERAS (ER arrival site) [112]. Depletion of Use1 or Ufe1 results in the lack of colocalization of Rab1 with TBSV p33 and greatly reduces viral accumulation [112,113]. Like Rab1, Ufe1 and Use1 are co-opted into TBSV VROs via interactions with p33 [113]. As the regulators of the secretory pathway, the SNARE proteins are also hijacked by other viruses for VRO formation. In the case of TuMV, the ER SNARE protein Syp71 colocalizes with the chloroplast-bound 6K2 complex. Downregulation of Syp71 inhibits TuMV accumulation and the formation of 6K2-induced chloroplast-bound elongated tubular structures and chloroplast aggregates [72]. In infection by *Cowpea mosaic virus* (CPMV, *Comovirus*), the SNARE-like ER protein Vap27-1 interacts with the CPMV 60K membrane protein and colocalizes with the 60K-induced membranous vesicles [114]. Recently, two SNARE SYP2 family proteins, SYP22 and SYP23, have been shown to interact with the TMV 126 kDa replication protein [115]. The TMV 126 kDa protein targets the ER and induces cytoplasmic bodies. It is not surprising that these SYP2 family proteins are required for normal TMV accumulation and spread [115]. 

#### 3.3.3. Endocytic and Recycling Pathway Components

An increasing body of evidence supports the involvement of endocytic pathway regulators in viral replication. Dynamins are important regulators of clathrin-mediated endocytosis (CME), which is a predominant endocytosis pathway in plant cells [116]. Recently, we have demonstrated that dynamin-related proteins DRP1 and DRP2 are host factors for potyvirus infection [117,118]. DRP1 and DRP2 interact with TuMV 6K2, VPg, and CI, essential for TuMV replication. In TuMV-infected cells, DRP1A, DRP2A, and DRP2B are recruited to VROs. Overexpression of DRP1A and DRP2 promotes TuMV replication, while knockdown or knockout of DRP2A or DRP2B in Arabidopsis suppresses TuMV replication. Treatment with a dynamin-specific inhibitor disrupts endocytosis and inhibits virus replication. These data suggest that TuMV co-opts DRP1 and DRP2 for VRO assembly [117,118].

Retromer plays a key role in the recruitment of cargo for endosomal trafficking and recycling [119]. It contains two subcomplexes: a core heterotrimer, composed of three vacuolar protein sorting-associated (VPS) proteins Vps26, Vps29, and Vps35, that engages and concentrates cargo for transport, and a membrane-associated sorting nexin (SNX) dimer that binds to endosomal membranes and mediates the formation of tubulovesicular carriers [120]. The retromer complex has been shown to be involved in tombusviral VRO synthesis [121]. The TBSV p33 and CIRV p36 proteins interact with the trimeric VPS complex and re-target the retromer cargoes into VROs rather than Golgi bodies, their canonical destination. This retargeting facilitates VRO biosynthesis through the delivery of several lipid synthesizing and modifying enzymes, such as Psd2 phosphatidylserine decarboxylase, Vps34 phosphatidylinositol 3-kinase (PI3K), and phosphatidylinositol 4-kinase (PI4Kα-like) [121]. In addition, with the help of the retromer complex, the endosomal sorting nexin protein Vps5 (SNX1 and SNX2a/b in plant), can also be delivered into VROs via its interaction with TBSV p33. As a permanent component of TBSV VROs, Vps5 contains a PI(3)P (phosphatidylinositol-3-phosphate) binding PX domain and a positive curvature-inducing BAR domain, and both domains are required for efficient TBSV replication. It has been suggested that Vps5 binds to the PI(3)P-rich portion of VROs to stabilize VROs and possibly the neck structure within the TBSV-induced spherules [122]. 

Early and late endosomes participate in VRO biosynthesis too. For example, endosomal Rab5 small GTPase (Vps21, Ypt52, and Ypt53 in yeast) is a key regulator of early endosomal biogenesis, maturation, trafficking, and membrane fusions. TBSV recruits Rab5 into VROs via the interaction with TBSV p33 [123]. Phosphatidylethanolamine (PE), a major class of phospholipids in biological membranes, is rich in the early endosomes. The p33-mediated recruitment of Rab5-associated early endosomal membranes enriches PE in TBSV VROs to support viral replication. Different from TBSV that targets endosomes, CIRV co-opts Rab5 into VROs on mitochondria [123]. In addition to Rab5, tombusviruses also recruit the endosomal small GTPase Rab7 for the formation of VROs [124]. TBSV p33 interacts with and targets Rab7 to the large VROs. It has been suggested that Rab7 facilitates the recruitment of the retromer complex, sorting nexin-BAR proteins, and lipid enzymes into VROs to create an optimal milieu for virus replication [124].

#### 3.3.4. Lipids and Sterols

Lipids are essential components of cellular membranes and directly affect membrane asymmetry, curvature, and proliferation [50,125]. Plant +ssRNA viruses manipulate host lipids and lipid pathways for VROs biosynthesis [126]. Different viruses prefer specific sets of lipids, including sterols, glycerophospholipids, and sphingolipids [50]. As noted above, PE, a phospholipid rich in the early endosomes, is an important component of tombusvirus VROs [123,127]. PE enrichment at the sites of TBSV replication is achieved through the recruitment of endosomal Rab5 small GTPase. Apparently, BMV needs phosphatidylcholine (PC) for VRO assembly. BMV, via the interaction with 1a, co-opts the PE methyltransferase Cho2p that catalyzes synthesis to stimulate PC accumulation at the replication sites to support proper VRO formation and promote viral replication [128]. Blocking PC synthesis results in the formation of spherules with wider diameters and inhibition of viral replication. Another excellent example is phosphatidic acid (PA). PA has been shown to promote viral replication for several viruses. PAH1 encodes PA phosphohydrolase that converts PA to diacylglycerol and regulates phospholipid synthesis. Deletion of PAH1 leads to high levels of PA, proliferation and enlargement of the ER membrane, and extension of the nuclear membrane. In the yeast *pah1* mutant, TBSV switches from the peroxisome to the expanded ER membrane for VRO formation [129], whereas BMV targets the extended nuclear membrane for VRO synthesis [130]. Regardless of this difference, TBSV and BMV replication is significantly accelerated in this mutant. Like TBSV, RCNMV from the same *Tombusviridae* family induces VROs that contain PA-producing enzymes phospholipase Dα (PLDα) and Dβ (PLDβ) [131]. RCNMV p27 interacts with PA and the interaction enhances the viral replication by upregulating VRO assembly. Downregulation of PLDs reduces the production of PA and inhibits RCNMV replication, and exogenous application of PA enhances viral replication [131].

Sterols are unique among lipids as they have a multiple-ring structure. Similar to common lipids, sterols also play a role in viral replication [132,133]. TBSV p33 can bind to sterol directly [133], and modulates host vesicle-associated membrane protein (VAMP)-associated protein (VAP) Scs2p and the oxysterol-binding protein-related proteins (ORPs) to facilitate the formation of membrane contact sites, which leads to the enrichment of sterols at the viral replication sites [132]. Recent experimental evidence has shown that phosphatidylinositol-3-phosphate (PI3P) and phosphatidylinositol-4-phosphate (PI4P) are crucial for TBSV replication as well [134,135]. TBSV hijacks Vps34 PI3K to generate PI(3)P, leading to PI(3)P enrichment in VROs [134]. Reduction in the PI(4)P level strongly inhibits TBSV replication [135]. For further information on the role of lipids and sterols in viral replication, readers are referred to several excellent in-depth reviews [50,126,136,137,138].

#### 3.3.5. Cytoskeleton

Accumulated evidence suggests that plant RNA viruses hijack the cytoskeleton for VRO biosynthesis. One such example is that TMV uses MTs for the anchorage of VROs and further development of these VROs into large virus factories [13,41,139]. TMV movement protein (MP) has a strong intrinsic affinity for binding to the ER and MT [140]. This binding activity contributes to the formation of ER-derived inclusions that harbor the viral factories. Therefore, TMV VROs are assembled at the cMERs, the three-way junctions of the cortical ER-actin network, and microtubules. These junctions function as cortical trafficking hubs where organelles and protein complexes exchange their components [141]. At late infection stages, many of the TMV MP-associated VROs remain attached to the cMERs, and grow into large virus factories by recruiting host factors and membranes provided by these junction sites [139]. In the case of TBSV, large VROs containing p33 are located close to actin patches in yeast or around actin cable hubs in plants [142]. TBSV subverts the actin network via the interaction of p33 with Cof1p, a major modulator of actin filament disassembly [142]. The p33-Cof1p interaction compromises the cofilin activities of this actin depolymerization factor and impairs the dynamics of the actin network. The stabilized actin filaments function as “trafficking highways” to ensure the efficient and rapid delivery of viral proteins and proviral host components required for VRO assembly [121,123,142]. For instance, through the subverted AFs, TBSV recruits Rpn11, a cytosolic protein interaction hub, to deliver cytosolic proteins, such as glycolytic and fermentation enzymes, to generate energy essential for VRO growth and viral replication [143]. Moreover, TBSV may also take advantage of the actin-assisted rapid biogenesis of VROs to limit the recruitment of cellular restriction factors (CIRFs) [144]. Therefore, for some RNA viruses such as tombusviruses, subversion of the actin network may serve as an effective strategy for the infecting virus to win the recruitment race through rapid delivery of proviral host factors into VROs and inhibition of cellular defense factors.

## 4. Plant Viruses Utilize the Endomembrane System and Cytoskeleton for Movement 

To establish systemic infection, plant viruses must cross the cell wall barrier via PD to enter and infect the adjacent cells. Viral cell-to-cell movement requires the co-ordinated action of viral MPs and the endomembrane system and associated cytoskeleton. For some plant RNA viruses, viral replication and intracellular movement are structurally and functionally linked processes [13]. This intracellular motility makes it possible to move the synthesis sites of progeny viruses in close proximity to PD for intercellular movement.

### 4.1. The ER Network 

Among eukaryotic living organisms, the plant ER is a unique structure that is continuous between adjacent cells via the PD desmotubules to form an ER network throughout the entire plant, and thus provides a direct pathway for the movement of ER-associated proteins or macromolecular complexes, including the viral MP-associated movement complex to PD and subsequently into neighboring cells. Indeed, TMV intercellular movement requires the ER membrane, but is independent of the secretory pathway. TMV MP is associated with the cytoplasmic face of the ER [145]. The intracellular trafficking of TMV MP and VROs is not sensitive to BFA treatment or Sar1 inhibition [146]. Treatment with high concentrations of BFA disrupts the ER and inhibits the PD targeting of the MP [147]. Some other plant RNA viruses, such as *Potato virus X* (PVX, *Potexvirus*) and *Poa semilatent virus* (PSLV, *Hordeivirus*) encoding triple gene block (TGB), seem dependent on the ER network, but not the secretory pathway for movement as well. TGB2 and TGB3 are small membrane-integrated proteins that are involved in the delivery of TGB1 and the viral genomic RNA bound by TGB1 to PD-associated sites for intercellular movement [148,149]. TGB3 is able to autonomously direct TGB2, TGB1, and virus-induced membrane structures to the PD [148]. Sar1 inhibition has no effect on the targeting of these membrane structures at the PD in viral infection [150]. 

The ER membrane transport system is also critical for the intercellular movement of the negative strand RNA *Tomato spotted wilt virus* (TSWV, *Tospovirus*) and its MP NSm [151]. TSWV MP NSm is physically associated with the ER membrane. Mutations in NSm that impair its association with the ER inhibit cell-to-cell trafficking. Pharmacological disruption of the ER network severely inhibits NSm trafficking. In the Arabidopsis mutant rhd3 with an impaired ER network, NSm-GFP trafficking and TSWV intercellular movement are significantly reduced [151]. The ER-to-Golgi secretion pathway and the cytoskeleton transport systems are not involved in the intercellular trafficking of TSWV NSm [151].

### 4.2. The ER-Golgi Secretory Pathway 

Unlike TMV, TSWV and viruses with TGB MPs discussed above, many other plant viruses require the ER-Golgi secretory pathway for cell-to-cell movement. *Grapevine fanleaf virus* (GFLV, *Nepovirus*) MP assembles tubules at PD with the assistance of PD-located proteins (PDLPs) for viral intercellular movement. Both GFLV MP and PDLPs localize to PD via the ER-Golgi secretory pathway, as disruption of the early secretory pathway by expression of the Sar1 dominant-negative mutant or genetic mutation of PDLP genes prevents the PD targeting of the GFLV tubule-forming MP [152]. TuMV intra- and intercellular movement are sensitive to the secretory pathway inhibitor BFA [153]. Endogenous application of BFA not only inhibits the intracellular movement of 6K2-labelled VROs but also prevents the PD targeting of TuMV P3N-PIPO and CI, two viral proteins essential for potyviral intercellular movement [71]. In the *Arabidopsis thaliana sec24a* mutant defective in the secretory pathway, TuMV systemic movement is delayed [154]. In addition to TuMV and GFLV, several other plant RNA viruses may also use the ER-to-Golgi secretory pathway for the PD targeting of viral MPs and subsequently, viral intercellular movement [155,156,157,158,159,160,161]. 

### 4.3. The Endocytic Pathway

The endocytic pathway appears to be involved in viral intercellular movement for several plant viruses from viruses encoding TGB MPs, tobamoviruses, and potyviruses. In the case of viruses with TGB MPs, the viral movement complex of *Bamboo mosaic virus* (BaMV, *Potexvirus*) moves from the ER membrane to the PM via the endosomal system by vesicle trafficking with the activation of NbRabF1, a plant-specific Rab5 small GTPase from *Nicotiana benthamiana* [162]. The two viral MP proteins TGB2 and TGB3 of *Potato mop-top virus* (PMTV, *Pomovirus*) traffic in the endocytic recycling pathway [163]. Synaptotagmins (SYTA) are calcium sensors that regulate synaptic vesicle exo/endocytosis. Expression of a dominant-negative SYTA mutant causes depletion of PM-derived endosomes and inhibits cell-to-cell trafficking of MPs from TMV and a DNA virus named *Cabbage leaf curl virus* (CaLCuV, *Begomovirus*) [164]. Moreover, SYTA also regulates the cell-to-cell movement of TuMV and *Turnip vein clearing virus* (TVCV, *Tobamovirus*) [164,165]. Recently, we have reported that endocytosis dynamin-like proteins interact with TuMV VPg and CI, which are essential viral proteins for potyvirus replication and intercellular movement [117]. Treatment with a dynamin-specific inhibitor disrupts endocytosis and the delivery of VPg and CI to endocytic structures, and inhibits viral replication and intercellular movement [117]. These viral proteins are recognized as cargoes by AP2β (adaptor protein complex-2β) and knockout AtAP2β interferes with VPg and CI endosomal trafficking [118]. CI interacts with AP2β through the acidic dileucine motifs, which are crucial for endosomal targeting and viral replication, suggesting that viral proteins bind to AP2β to mediate intracellular trafficking for viral replication [166].

### 4.4. The Cytoskeleton Transport System

Trafficking of organelles and virus-induced complexes in plant cells is largely dependent on the dynamics of the cytoskeleton [35,36,37]. The actomyosin mobility system, also known as the actin-myosin complex that forms within the cytoskeleton, is the primary force-generating machinery. The intact actin microfilament has been shown to be required for movement by several plant RNA viruses, such as TMV, PVX, TBSV, TuMV, TSWV, and *Barley yellow striate mosaic virus* (BYSMV, *Cytorhabdovirus*) [167,168]. Disruption of AFs with microfilament inhibitors latrunculin B (LatB) and cytochalasin D (CytD) significantly inhibits viral intercellular movement and abolishes the trafficking of VROs. Viral movements on actin filaments require myosin motor activity. In plants, myosins are grouped into two classes, class VIII and XI [169]. Specific myosins XI-2 and XI-K in class XI are recruited for the PD targeting of GFLV MP and PDLPs, tubule formation, and viral movement [170]. These two myosins are also important for TuMV movement [71,171]. The expression of a dominant-negative mutant of myosin XI-K or XI-2 inhibits the intracellular trafficking of TuMV 6K2 vesicles [71] and viral intercellular movement [171]. Myosin VIII-1 is required by the PD-targeting of the P7-1 protein of *Rice black-streaked dwarf virus* (RBSDV, *Fijivirus*) [160], and pC6 and NSvc4, encoded by the two tenuiviruses *Rice grassy stunt virus* (RGSV) and *Rice stripe virus* (RSV), respectively [157,161]. Different myosins may be recruited for specific steps in the process of TMV movement [172]. Myosins XI-2 and XI-K play specific roles in the intracellular movement of TMV by supporting the ER-associated transport, whereas myosins VIII-1, -2, and -B facilitate the movement by supporting the specific targeting of TMV MP to PD. 

The involvement of MTs in plant viral movement has also been studied in TMV [139,173]. MTs participate in guiding the trafficking of VROs along the ER/actin network [139]. The release of MP particles from the anchorage sites depends on MT polymerization [174]. Upon treatment with MT polymerization inhibitor APM (aminoprophos-methyl), the MP-associated particles remain stably anchored at the cMERs rather than detaching from these sites [174]. ER-mediated trafficking of VROs to PD also depends on MT polymerization [175]. In the tobacco mutants with reduced MT polymerization dynamics, the association of MP with PD and the cell-to-cell movement are reduced [175].

## 5. Conclusions and Future Prospects

**Figure 2:**
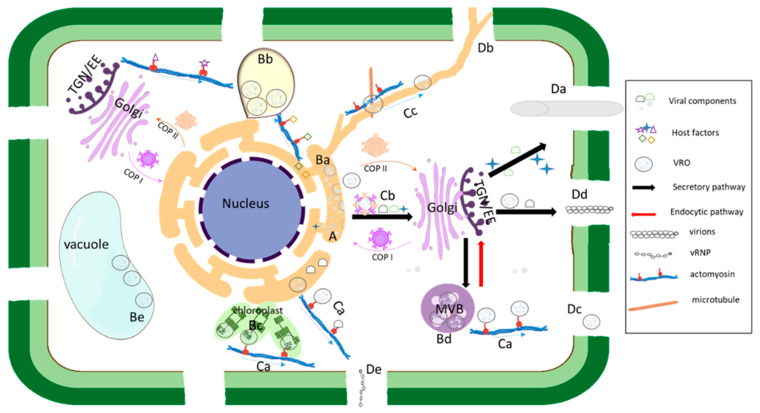
Schematic representation of the involvement of endomembrane system and cytoskeleton in viral replication and movement. Early in the infection process, translation of viral RNAs occurs at the rough ER (A). The viral membrane-targeting protein(s) target specific organelles such as the ER (Ba), peroxisomes (Bb), chloroplasts (Bc), multivesicular bodies (MVBs) (Bd) and vacuole (Be) and remodel organellar membranes to initiate the formation of viral replication organelles (VROs), and various virus-specific host proteins (host factors) are recruited to VROs. Dependent on virus, different cellular endomembrane-cytoskeleton network components, such as the ER, ER-Golgi, microtubules, actin filaments and endosomes, are hijacked to play roles in VRO biogenesis. VROs, viral movement protein(s) (MPs) and MP-associated viral movement complexes traffic to the plasmodesmata (PD) via the cytoskeleton machinery (mainly the actomyson mobility system) (Ca), secretory pathway (Cb), or the ER membrane (Cc). To pass through the PD, some viral MPs may form tubules within the PD to displace the desmotubule (Da) and others may increase the size exclusion limit (SEL) of PD (Db). Different viruses may pass through the PD via different MP-associated complexes: VRO (Dc), virion (Dd), or the viral ribonucleoprotein (vRNP) complex (De).

In this review article, we have briefly summarized the current knowledge about how plant viruses deploy their membrane protein to target preferred cellular organelles and further recruit various host factors to modify membranes for VRO biogenesis. We have discussed that some viruses have the ability to target alternative organelles for viral replication. The virus-induced cellular membranous structures are in different forms, such as spherules, vesicles, and MVBs. We have highlighted progress in the recruitment of the endomembrane system and cytoskeleton by plant RNA viruses to move intracellularly to PD for their intercellular movement (Figure 2). 

Despite the significant advances in the role of the endomembrane-cytoskeleton network in VROs biogenesis, many intriguing questions remain to be answered. For example, it has been shown that some ER-derived small virus replication structures are mobile at the early stage and form large static aggregated structures (VROs). The mechanical details underlying this transition are largely unknown. VRO intercellular movement is still an understudied area. It is not clear if VRO mobility is a conserved feature for all plant RNA viruses. What is the minimum requirement of the host factors for VRO biogenesis? Are any host factors conserved among different hosts and viruses? How do VROs concentrate essential materials, including energy supplies, for viral replication? Moreover, as always, novel discoveries lead to more questions. For example, a recent study has shown that a dsRNase with dsRNA-degrading activity encoded by the broad-spectrum SMV *R* gene *Rsv4* can target SMV VROs to inhibit viral multiplication [176]. This work suggests that some plants may have evolved antiviral mechanisms by directly targeting VROs, challenging the protective role of VROs during viral replication. 

The molecular identification and functional characterization of host factors essential for viral replication and movement are of great interest, as host factors may be targeted for the development of genetic resistance in plants through advanced technologies such as the precise genome editing technology. Effective methods for the discovery of host factors, such as proximity labeling [177], lipidomic approaches [178], and in vitro reconstitution of VROs using purified proteins, viral RNA and artificial liposomes or giant unilamellar vesicles [5,179] will be increasingly valuable. High-throughput profiling of purified VROs may provide a full view of the host components of VROs and facilitate dissecting the molecular mechanisms underlying the biogenesis of VROs. In addition, the latest powerful imaging techniques, such as cryo-electron tomography subtomogram averaging and classification (cryoSTAC) [180], can assist in investigating the structural details of VROs, and quantitative live-cell and super-resolution microscopy [181] can allow for the spatio-temporal analysis of viral infection in living cells. Ultimately, these studies will not only advance knowledge in plant virology and virus-plant interactions, but also assist in the development of novel effective antiviral strategies for sustainable crop production.
